# Supplementary material for: Development of the parental self-efficacy scale for preventing challenging behaviors in children with autism spectrum disorder
Source: PLoS One. 2020 Sep 3;15(9):e0238652. doi: 10.1371/journal.pone.0238652 (PMC7470344; doi:10.1371/journal.pone.0238652)
Supplement: S1 Appendix — (PDF) [file pone.0238652.s001.pdf]

S1 Appendix. PASEC English Version

Parental Self-Efficacy Scale for Preventing Challenging Behaviors in Children with Autism Spectrum Disorder (PASEC), English version

Please circle (○) the **number** that is the closest to your thought for each statement.

Evaluation
0: disagree.
1: slightly disagree.
2: slightly agree.
3: agree.

No	domain & item	evaluation			
**Empowerment of children's sociality**					
1	I can communicate to my child that I am keeping a caring eye on him/her.	0	1	2	3
2	I can communicate to my child that I sympathize with him/her.	0	1	2	3
3	I can ascertain what my child wants to do.	0	1	2	3
**Optimization of children's environment**					
4	I can create places where my child feels comfortable.	0	1	2	3
5	I can reduce stimulations that my child does not like.	0	1	2	3
6	I can create opportunities for my child to interact with people in a way that is appropriate for his/her growth and development.	0	1	2	3
	No.1-6 total	point			

Kabashima Y, Tadaka E, Arimoto A: Development of the Parental Self-Efficacy Scale for Preventing Challenging Behaviors in Children with Autism Spectrum Disorder, PLOS ONE, 2020.
