# Supplementary material for: Development of the parental self-efficacy scale for preventing challenging behaviors in children with autism spectrum disorder
Source: PLoS One. 2020 Sep 3;15(9):e0238652. doi: 10.1371/journal.pone.0238652 (PMC7470344; doi:10.1371/journal.pone.0238652)
Supplement: S2 Appendix — (PDF) [file pone.0238652.s002.pdf]

S2 Appendix. PASEC Japanese Version

Parental Self-Efficacy Scale for Preventing Challenging Behaviors in Children with Autism Spectrum Disorder (PASEC), Japanese version

自閉症スペクトラム児のチャレンジング行動予防に向けた親の自己効力感尺度(PASEC), 日本語版

各項目について、あなたの考えとして最も近いと思う数字１つに○をつけてください。

領域　／　項目	0<br>そう思わない	1<br>やや<br>そう思わない	2<br>やや<br>そう思う	3<br>そう思う
**児の社会性の促進**				
1　私は、子どもに見守っていることを伝えられる	0	1	2	3
2　私は、子どもの気持ちに共感していることを伝えられる	0	1	2	3
3　私は、子どもの意思を確認することができる	0	1	2	3
**児の環境の最適化**				
4　私は、子どもが落ち着けるように居場所を工夫できる	0	1	2	3
5　私は、子どもの苦手な刺激をやわらげられる	0	1	2	3
6　私は、子どもの成長・発達に合った人とのふれあいの機会を作ることができる	0	1	2	3
No.1-6　合計	点			

Kabashima Y, Tadaka E, Arimoto A: Development of the Parental Self-Efficacy Scale for Preventing Challenging Behaviors in Children with Autism Spectrum Disorder, PLOS ONE, 2020.
